# Changes in selected physicochemical properties of lysozyme modified with a new method using microwave field and oxidation

**DOI:** 10.1371/journal.pone.0213021

**Published:** 2019-02-28

**Authors:** Tianyu Yang, Grzegorz Leśnierowski

**Affiliations:** Food Quality and Safety Management, Poznan University of Life Science, Poznan, Poland; Brandeis University, UNITED STATES

## Abstract

Lysozyme is a type of enzymatic protein found in a wide range of organisms. Among the many applications of lysozyme, the antibacterial activity features caused by the hydrolysis of 1–4 glycosidic bonds between N-acetylmuramic acid and N-acetylglucosamine of gram-positive bacteria are beneficial in the food industry, medicine, trade, and pharmacology. Studies have indicated that specific modifications of lysozyme cause oligomerization of the enzyme, and the resulting dimer, which also undergoes changes in physicochemical properties, shows greater total antibacterial activity. Among these modifications, thermo-chemical methods are one of the most important groups. In this study, the microwave method of the enzyme heating with the associated process of enzyme oxidation was used as a novel thermo-chemical method to induce lysozyme oligomerization. The research shows that using this new method can produce enzymatic preparations composed of approximately 58.9% oligomers, including 33.5% dimer and 25.4% trimer under a hydrogen peroxide concentration of 4% and pH of 8. The maximum percentage of lysozyme dimer of 39.4% was obtained at pH 6.0 with the addition of 2% oxidant. In addition, as a result of the modification process, the hydrolytic activity and surface hydrophobicity of the enzyme were changed.

## Introduction

Lysozyme is a small molecule enzyme (14 400 Da). Its antibacterial activity is the original natural mechanism for defense against bacteria in humans, animals, and plants and is a large component of the eggs. The action of lysozyme is based on hydrolysis of the β-1, 4-glycosidic linkage between N-acetylmuramic acid and N-acetylglucosamine, which present in the cell walls of many bacteria. Unfortunately, the antibacterial action of the enzyme only applies to gram-positive bacteria. Gram-negative bacteria are resistant to its effects [1–2]. Many studies have shown that this limitation can be changed by various types of lysozyme modifications, such as chemical, physical and genetic changes that improve its properties and stability [3]. The lysozyme protein structure is maintained by four disulfide bonds [4, 5]. Modifications of lysozyme cause isomerization of these bonds and other structural change, resulting in oligomerization of the enzyme, changes in its hydrophobic surface and the emergence of new antibacterial activity also directed against gram-negative bacteria [2, 6].

To date, several original methods have been developed to modify lysozyme. Research has focused mainly on thermal and chemical modifications of the enzyme, in low-, medium- and high-temperature ranges. Membrane techniques have also been used for this purpose [2, 7–9]. The preparations formed as a result of these modifications showed greatly improved antimicrobial action towards gram-negative bacteria. Additionally, lysozyme modified to oligomeric forms shows many other useful properties. The enzyme in the dimer form modulates the synthesis of tumor necrosis factor (TNFα) and stimulates the production and release of *interferon alpha* and gamma (INFα, INFγ) as well as interleukin-2 (Il-2) and interleukin-6 (Il-6) by human lymphocytes [2, 10–13].

Seeking more effective ways to modify lysozyme, the purpose of this work was to introduce a new method of lysozyme modification based on the use of microwave field interaction with chemical processes and to evaluate selected physicochemical properties of the obtained preparations. Our previous research indicates that such a modification system can provide a very good result.

## Method and material

### Materials

The experimental material was lysozyme monomer isolated from chicken egg white with a hydrolytic activity of 21 252 U/mg, as produced by the Belovo company (Bastogne, Belgium).

### Modification of lysozyme

The modification was carried out in two stages: A-modified by a microwave field and B-chemical modified by oxidation.

For both modifications, lysozyme was prepared as a 5% (w/V) water solution. The solution was adjusted to a pH of 4.0, 6.0, and 8.0 using 1 M NaOH or 1 M HCl, respectively and then 15 ml of each solution was placed in sealed conical flask, numbered containers (5 replicates for each pH level).

A-modified by microwave field:

The modification by a microwave field was carried out in a microwave device (magnetron manufactured by Sharp, model R-879-A) under the following conditions:

- microwave power—270 watts.
- time of modification—3 minutes.

After this process, the samples were immediately cooled in ice water.

B-The chemical modification was carried out immediately after the microwave modification. This process consisted of oxidation of the previously modified enzyme with hydrogen peroxide at the following concentration in the samples: 0%, 1%, 2%, 3%, and 4%. This process lasted 24 hours, after which the samples were frozen and lyophilized in a Labconco freeze dryer (USA). The operation conditions of the lyophilization were 0.022 mbar, -54°C and drying to obtain samples with a water content of 7–8%.

The obtained preparations were subjected to analysis of the base chemical properties, the degree of enzyme oligomerization, hydrolytic activity and changes in the surface hydrophobicity of the enzyme.

### Analytical procedures

#### Electrophoresis

According to a previously published method [5, 14], the content of the oligomer was determined by using an SE-600 electrophoresis apparatus. The electrophoresis was carried out on an acrylamide gel containing 12% acrylamide separating gels and 6% stacking gels with 0.1% SDS. The samples prepared in the sample buffer (consisting of 0.3 M TRIS-HCL (pH 6.8), 30% glycerol, 0.1% bromophenol blue and 6% SDS) were heated at 100°C for 5 minutes and then applied to the gel in amounts of 10 μl. The electrophoresis was performed under a 60 mA current in the stacking gel and 90 mA current in the separating gel for approximately 5 hours.

After the electrophoresis process, the gels were fixed in a solution of acetic acid, methanol, and water for 1 hour and stained with 0.025% Coomassie brilliant blue R solution for 20 hours. Finally, the gels were discolored in diluted acetic acid until a transparent background obtained.

The amounts of oligomer in individual samples were calculated with the TotalLab Quant software (Nonlinear Dynamics Ltd., Durham, NC, USA).

#### Hydrolytic activity of modified lysozyme

Lysozyme hydrolytic activity was determined by the use of the spectrophotometric method, which based on the phenomenon of bacterial cell wall lysis by the enzymes [15, 16]. The lytic activity of lysozyme was determined by monitoring the decrease in turbidity of a suspension of *Micrococcus lysodeikticus* (Sigma-Aldrich, St. Louis, USA) cells at 450 nm. The activity presented as the rate of decrease in absorbance per minute (Δ abs/min).

#### Hydrophobicity of modified lysozyme

The hydrophobicity of the modified lysozyme was determined according to the research [17]. This method involves the spectrophotometric measurement ability of the tested protein binding with the detergent by determining the shade of these two substances mixture solution after adding dye. The ability was related to the hydrophobicity of the protein. A 0.1% solution of modified preparations were prepared, using 0.25% Tween-80 detergent dissolved in deionized water. A 20% solution of Bio-Rad dye made in methanol which was diluted 10-fold with water and filtered through a soft filter. The tested tubes were labeled a’, b’, a, and b. In tubes a and a’, 50 μl of the modified solution was applied to the bottom. Tubes b and b’ were applied with 50 μl of water. a’ and b’ were treated with 50ml Tween-80 solution. Each separate tube was analyzed at λ = 595 nm, and the hydrophobicity was calculated with the following formula:

$$\mathrm{PH}=\frac{\left[a-b\right]-\left[a^{\prime }-b\prime \right]}{\left[a-b\right]}*100\%$$

#### Statistical analysis

The test results were analyzed using the STATISTICA software for correlation, dependent regression analysis and one-way analysis of variance (ANOVA).

## Result and discussion

The method of lysozyme modification presented in this work is a combination of thermophysical and chemical processes. For the thermophysical process, we chose the energy of the microwave field. Generally, the use of microwaves in chemical technology is not new and is even widely applied. There are also a few examples of the using of microwaves as an auxiliary factor in enzyme chemistry with precise control of the application parameters [18]. The novelty here is the attempt to use this energy source to modify lysozyme. The microwave technology heats the enzyme via the action of heating the aqueous medium. This action is necessary for the thermal modification of lysozyme, and therefore, we assumed that this technology has the potential to become an alternative way to modify the enzyme. For the second modification, we chose a chemical process based on enzyme oxidation of lysozyme, which we have used earlier for this purpose [7, 19]. We assumed that the combination of these specific processes would provide the expected effects, i.e., a high degree of oligomerization of lysozyme and improvement of its other properties. The purpose of this work was to use this new method in practice and to assess, on the basis of selected physicochemical properties, how much the enzyme was changed.

**Fig 1:**
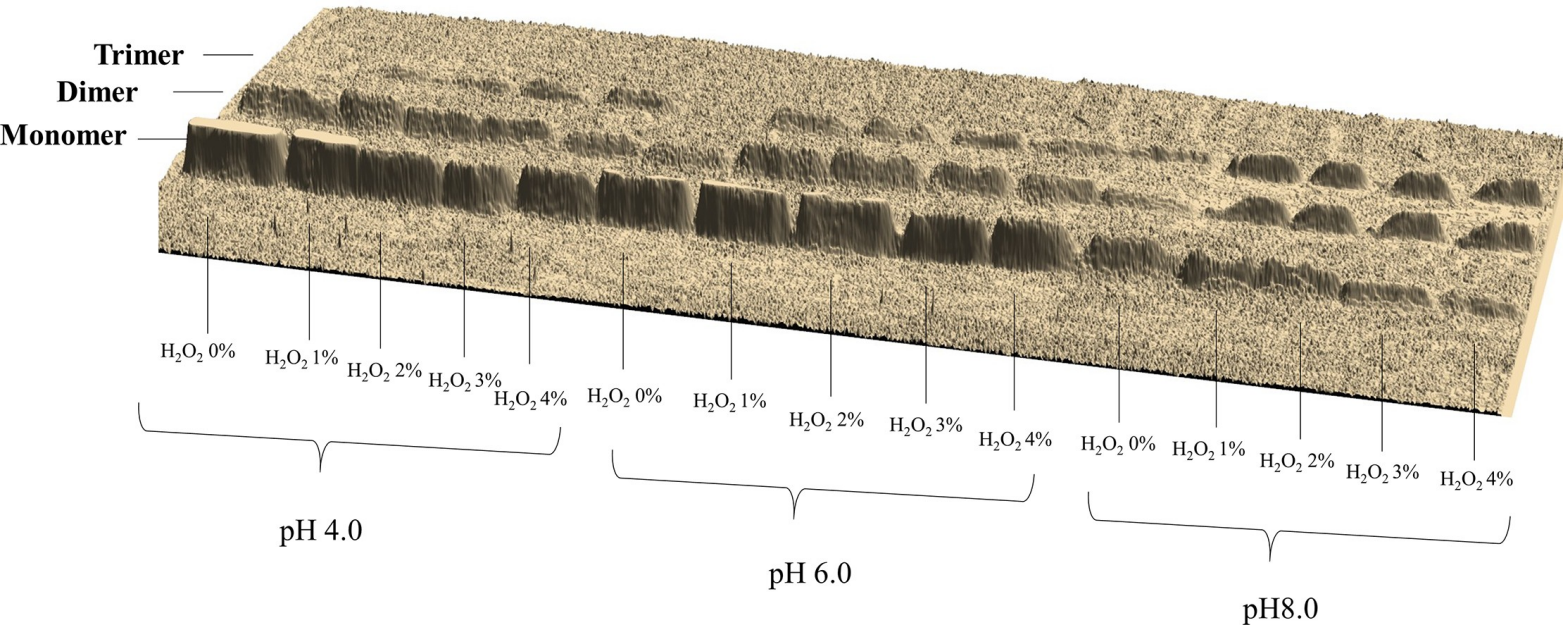
Electrophoretic image of modified lysozyme.

After modification carried out in accordance with the procedure given in paragraph 3.2, we obtained preparations of modified lysozyme (Fig 1) that were analyzed as described in section 3.3. All results for these samples are presented in Table 1.

**Table 1:** Characterization of the lysozyme modified with a combination of a microwave field and oxidation.

No.	PH	H_2_O_2_ (%)	Dimer content (%)	Trimer content (%)	Total content (%)	Hydrolytic activity (U/mg)	Change in surface hydrophobicity (ΔH)(%)
1	4	0	17.1	0.0	17.1	19720	18.22
2	4	1	28.8	8.4	37.2	10440	22.45
3	4	2	30.2	12.4	42.6	6300	30.28
4	4	3	33.4	15.6	49.0	5000	35.80
5	4	4	36.4	17.7	54.1	4320	40.11
6	6	0	17.4	0.0	17.4	15240	23.44
7	6	1	31.5	10.9	42.4	9025	29.92
8	6	2	32.3	13.2	45.5	7600	37.18
9	6	3	35.2	15.5	50.7	4120	40.95
10	6	4	38.5	17.9	56.4	3900	45.12
11	8	0	18.3	6.1	24.4	10200	30.46
12	8	1	24.4	20.1	44.5	3350	35.48
13	8	2	28.3	23.3	51.6	2750	45.09
14	8	3	30.2	24.5	54.7	2200	49.92
15	8	4	33.5	25.4	58.9	1675	54.48

**Fig 2:**
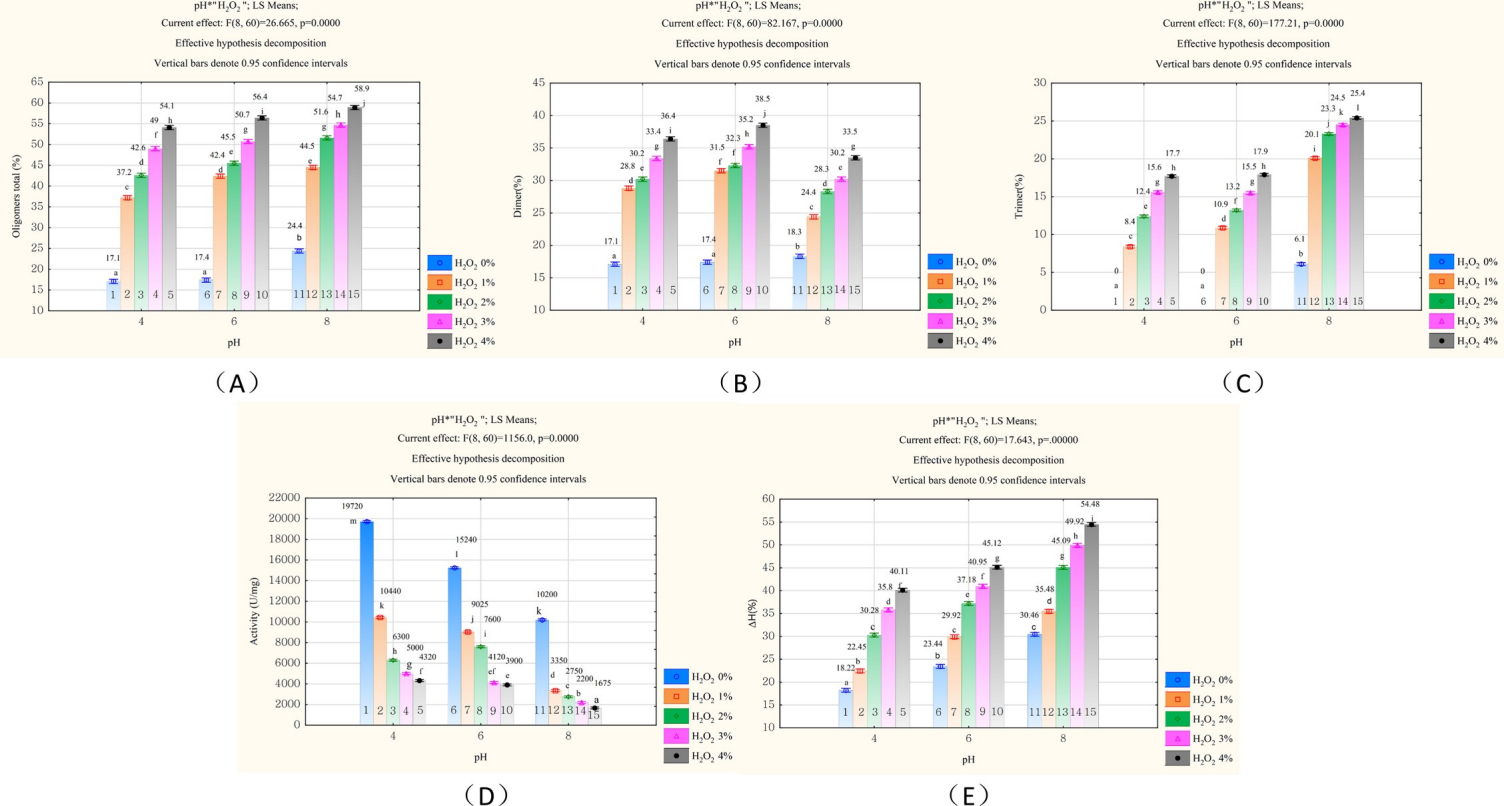
**Effect of lysozyme concentration at pH 4.0, 6.0, 8.0 and H**_**2**_**O**_**2**_ **concentration (0~4%) on (A) total oligomer, (B) concentration of dimer, (C) concentration of trimer, (D) activity of lysozyme samples, and (E) hydrophobicity** (a-l different superscripts in the separate bar denote a statistically significant difference at p ≤ 0.05).

The results for samples 1, 6, and 11 **(Fig 2A–2C)** showed that the microwave modification alone gave good results. At pH 4 and 6, a composition of more than 17% dimer was obtained (sample 1 and 6), and when the pH was 8, the composition was approximately 18% dimer and 6% trimer, producing over 24% oligomers in total (sample 6). However, when the oxidation modification was added, a significant increase in the resulting level of oligomers was observed (Tab. 1). Depending on the acidity of the environment and the quantity of oxidant, the level of oligomers increased to almost 60%, in which the volume of dimer and trimer increased significantly. In addition, we observed that the microwave modified lysozyme was completely dissolved in aqueous solutions every time. Other thermal methods gave a similar degree of oligomerization, but the solubility of the product was definitely lower and reached a maximum of 80% in the best samples [1, 6]. Besides, our new method of enzyme modification took less time and was easier to carry out than other thermal methods. High total solubility of preparations and high remaining hydrolytic activity facilitate their practical use [20]. Thus, our results indicate that the microwave field is a very good lysozyme modifier, and the proposed method can be an excellent alternative to the established methods.

According to Fig 2, this new method of lysozyme modification based on the use of microwave field energy and enzyme oxidation intensifies the formation of oligomers with increasing pH of the environment. At the same time, the amount of added oxidant had a significant impact on this effect. The observed trend, which was statistically significant, showed that the total amount of oligomer, including dimer and trimer, increased with increasing pH value and with an increasing concentration of added oxidant. The highest value of oligomer (24.4% to 558.9%) was obtained when the pH was 8. In contrast, the proportion of oligomers obtained at pH 4.0 was the lowest and ranged from 17.1 to 54.1%. These values varied with the amount of oxidant, which changed from 0 to 4%. Nevertheless, it should be not ignored that the maximum proportion of dimer (24.4% to 33.5%) formed in the higher oxidant concentration (2% to 4%) under pH 8.0 was significantly lower than the proportion of dimer (31.5% to 38.5%) formed under pH 6.0. The amounts of trimer increased, and the formation of dimer was simultaneously inhibited with an increase in pH above 6.0. However, in previous investigations, researchers have raised that the dimer has the greatest effect on the antibacterial action of lysozyme [21, 22]. A higher pH in this method can produce more oligomers. At the same time, a higher pH can also reduce the amount of dimer, which maybe has negative effects on the antibacterial function of the modified lysozyme.

We also conducted a univariate analysis of variance and linear regression of the proportion of oligomer with using the Statistica software. Different factor effects are shown in Tables 2 and 3.

**Table 2:** Univariate tests of significance for total oligomers performed with Sigma-restricted parameterization and effective hypothesis decomposition.

Effect	SS	Degrees of Freedom	MS	F	P
Intercept	139320.8	1	139320.8	422475.7	0.00
pH	595.8	2	297.9	903.4	0.00
H_2_O_2_	12215.6	4	3053.9	9260.7	0.00
pH* H_2_O_2_	70.3	8	8.8	26.7	0.00
Error	19.8	60	0.3		

**Table 3:** Regression of the dependent variables for total oligomers.

Effect	b*	Std. Err. of b*	b	Std. Err. of b	t (72)	p-value
Intercept			16.11667	2.341883	6.88193	0.000000
pH	0.212284	0.043957	1.70500	0.353052	4.82932	0.000007
H_2_O_2_	0.903224	0.043957	8.37667	0.407669	20.54769	0.000000

R = 0.92783490, R^2^ = 0.86087760, Adjusted R^2^ = 0.85701309, F (2, 72) = 222.76 p

b* is the Standardized regression coefficient.

b is Non-standardized regression coefficient

The pH and concentration of H_2_O_2_ had a significant effect on the formation of oligomer, including dimer. Moreover, the interaction between these two factors was also a determinant of the transformation of monomers into oligomers.

In addition, according to Table 3, the effects of both factors on the oligomers formed were shown with this formula:

Oligomers=16.1167+1.706x+8.3767y

The coefficient (8.3767) of this formula suggested that H_2_O_2_ level had a greater effect on the level of monomers than the pH change under the microwave heat modification. The R2 value (R^2^ = 0.8608776) of the equation approaching 1 means the goodness of fit of the formula is high.

Furthermore, our research showed that in addition to intensive oligomerization, the microwave and oxidative modification of lysozyme also changed the hydrolytic activity and hydrophobic surface of the enzyme (Table 1, Fig 2). Statistical analysis showed that with the increase in the oligomer formation, the surface hydrophobicity increased significantly, while the hydrolytic activity of the enzyme decreased (Fig 2). The hydrophobicity increased to a maximum of approximately 54.5% compared to the unmodified enzyme. Meanwhile, the hydrolytic activity decreased from 19720 U/mg to 1675 U/mg. Despite the lower enzyme activity during the modification process, compared to other methods of thermal modification of lysozyme, this method retained more natural hydrolytic enzyme activity at similar pH and produced higher levels of oligomers [7, 23]. Even under ideal conditions, the ordinary high-temperature modification method can reduce the enzyme activity to 1000 U/mg or lower [23]. However, from previous studies, it is known that despite the decrease in hydrolytic activity, the modified lysozyme shows even stronger bactericidal action, including against gram-negative bacteria. The effect of the modification is a new activity directed against gram-positive and gram-negative bacteria, and this novel activity consists of the remaining part of the hydrolytic activity and completely new activity, the origin of which is not yet explained [21,22]. This phenomenon is currently being intensively studied. The Japanese researcher Ibrahim hypothesized that this new activity is closely related to the increase in the surface hydrophobicity of the enzyme after its modification [24, 25]. He concluded that the new activity was closely related to the conformation of the dimer and the surface hydrophobicity of the enzyme. Amphiphilicity and the surface side chain cluster of the dimer are conducive to the combination of lysozyme and bacterial membrane because of theβ-sheets construction of dimer [24]. This properties give a strong bactericidal effect on gram-positive and gram-negative bacteria. Cegielska-Radzieiewska [22] also found a positive correlation between the content of dimer and the antibacterial ability of *P*. *mirabilis* in their thermochemical modification experiments. In addition, the cluster of hydrophobic side chain formed out a hydrophobic patch of solvent accessible surface area that lead to modified lysozyme interactions with bacteria membranes and penetration of bacteria [4, 26, 27]. The results shows that the bactericidal activity of lysozyme is in direct proportion to the hydrophobicity and in inversely proportion to the enzyme activity in his thermal modification experiment. In another experiment, a synthetic DNA fragment encoding of hydrophobic pentapeptide inserted into the wild-type lysozyme[28]. And the author showed that the antimicrobial effect against gram-negative bacteria of modified lysozyme appeared and was strengthened in spite of 75% of the lytic activity of wild-type lysozyme.

In our research presented in this work, we do not analyze the effects of the preparations on different types of bacteria, but we can expect that the effects will be similar to those described by Ibrahim [29] and other researchers [21, 22].

In conclusion, taking into account the results of research and their analysis in relation to the current state of knowledge, we can state that to obtain the best antibacterial effect in this study, i.e., the largest effect of enzyme dimerization, the more suitable for application lysozyme modification conditions by this new method is an environment with a pH of 6.0 and an addition of 4% oxidant. Although dimerization is positive correlates with the concentration of hydrogen dioxide. However, the higher amount of oxidants can pose difficult applications in the food and pharmaceutical industries and safety threat to consumers. Hence, 4% of the concentration of H_2_O_2_. is the amount we recommend. The optimal treatment conditions still need to be explored in future studies. Evaluation of the complete antimicrobial effect of the microwave-modified lysozyme, separate studies are being conducted now. These results of which will be the subject of our next work.

## Conclusion

This study indicates that microwave thermal modification of lysozyme can increase the formation of oligomers in comparison to conventional physical heating, improving the enzyme’s hydrophobicity by the use of this novel heat source for physicochemical modification; in addition, the method has the advantage of improving the modified lysozyme powder with faster and easier operation. Three minutes of heating can achieve a good result. The solubility of preparations reached 100%, making them easier to use than the preparations from conventional thermal methods with a solubility of 50–80%. The mixing method was very simple and compact. This method requires fewer technological devices, common chemicals, and a suitable device for manufacturing a microwave field.

The next steps of research should address the microwave processing time of the microwave modification and microbiological tests. This investigation described the relationship between microwave modification and antimicrobial activity in the mixer modification of lysozyme.

## Supporting information

S1 Data S1 Data. (XLSX)
